# Tobacco endgame goals and measures in Europe: current status and future directions

**DOI:** 10.1136/tc-2024-058606

**Published:** 2024-06-17

**Authors:** Hanna Ollila, Otto Ruokolainen, Tiina Laatikainen, Helena Koprivnikar, and JATC-2 WP9 co-authors, Adrián González-Marrón

**Affiliations:** 1 Department of Public Health, Finnish Institute for Health and Welfare, Helsinki, Finland; 2 Institute of Public Health and Clinical Nutrition, University of Eastern Finland, Kuopio, Finland; 3 National Institute of Public Health of the Republic of Slovenia, Ljubljana, Slovenia

**Keywords:** Public policy, Prevention, End game, Tobacco industry

## Abstract

The European Union (EU) aims for a tobacco use prevalence of less than 5% by 2040 with its Tobacco-Free Generation goal, aligning with the tobacco endgame approach. In the Joint Action on Tobacco Control 2 (JATC-2) -project, we examined adopted and planned endgame goals and measures as well as preparedness to counter tobacco industry interference in the process. We surveyed key informants in 24 out of 50 countries in the WHO European Region (19 of the 27 EU Member States, MS). Altogether, eight countries (7 EU MS) had official governmental endgame goals, and an additional six EU MS had similar proposals from government, civil society or research entities. Movement towards tobacco endgame was most evident in retail-oriented and consumer-oriented policies. These include restricting the sales of tobacco and related products and raising the age limit above 18 years. Product standards were used especially to regulate flavours but no measures to substantially reduce addictiveness were reported. Market-oriented measures that tap into industry profits were predominantly missing, and countries often lacked concrete tools to prevent industry interference. Respondents’ concerns around tobacco endgame were related to high smoking prevalence in some population groups, non-combustible and new nicotine products, cross-border marketing, political will, challenges with the existing regulations and industry interference. Results indicate both momentum and challenges in adopting and disseminating measures that facilitate achieving tobacco endgame goals. The EU goal can be used to advocate for national endgame goals and measures, and for the strengthened implementation of the WHO Framework Convention on Tobacco Control.

**WHAT IS ALREADY KNOWN ON THIS TOPIC**

- In the tobacco endgame approach, the focus is shifted from controlling the tobacco epidemic to ending it by reducing use to a minimal level in the population with structural, political and social changes. In the European Union, this is supported by the recently launched Tobacco-Free Generation goal.
- Tobacco endgame is well aligned with the WHO Framework Convention on Tobacco Control, which encourages parties to implement measures beyond the convention to better protect human health and obliges them to adopt effective measures to prevent and reduce nicotine addiction besides tobacco consumption.

**WHAT THIS STUDY ADDS**

- While several European countries already have governmental tobacco endgame goals or proposals towards these, there is substantial variation in their definitions, timelines and coverage of tobacco and nicotine products.
- Adopted and planned tobacco endgame measures centre around product-oriented, retail-oriented and consumer-oriented policies, such as product standards to reduce appeal, restricting sales and increasing the age limit above 18 years.

**HOW THIS STUDY MIGHT AFFECT RESEARCH, PRACTICE OR POLICY**

- Knowledge sharing facilitates the dissemination of tobacco endgame approach.
- More focus is needed on measures that can be expected to have a substantial impact on product availability, appeal and addictiveness.
- Concrete tools to prevent and counter tobacco industry interference are needed, as it is seen as a clear challenge in tobacco endgame.

## Introduction

 In the WHO European Region, encompassing 53 countries including 27 European Union (EU) Member States, 25% of adults use tobacco.^1^ This prevalence ranks second highest among the WHO regions, with a relatively slow decline compared with other regions. In 2021, as part of Europe’s Beating Cancer Plan, the EU announced a ‘Tobacco-Free Generation’ goal for the region.^2^ While the concept of tobacco-free generation originates in a proposal to limit tobacco sales by year born,^3^ the EU goal is defined as less than 5% of the population using tobacco by 2040. The EU goal aligns with the tobacco endgame approach, where the focus shifts from controlling the tobacco epidemic to ending it by reducing use to a minimal level in the population with structural, political and social changes.^4^ The EU goal is well justified under the WHO Framework Convention on Tobacco Control (WHO FCTC), which encourages parties to implement measures beyond the convention to better protect human health (Article 2.1) and obliges them to adopt effective measures and cooperate in developing appropriate policies to prevent and reduce tobacco consumption, nicotine addiction and exposure to tobacco smoke (Article 5.2b).^5^ Several European countries have already set their national tobacco endgame goals prior to the EU goal.^6^ We examine the current status of adopted and planned national goals and measures in the WHO European region, and how these reflect the EU goal among the Member States. We also examine how experts perceive the likelihood of adopting or achieving the endgame goal in their own country, and countries’ preparedness to counter tobacco industry interference in the process.

## Methods

In the Joint Action on Tobacco Control 2 (JATC-2) project,^7^ Work Package 9 (WP9) is tasked to identify national tobacco endgame strategies and forward-looking tobacco control policies, to explore and exchange best practices in the development, implementation and evaluation of these strategies and policies, and to facilitate their development in the European region. The WP9 involves 21 partner organisations from 15 European countries (Belgium, Cyprus, Denmark, Finland, France, Greece, Hungary, Ireland, Italy, Lithuania, Norway, Portugal, Serbia, Slovenia and Spain), with the Netherlands collaborating. As part of this work, we surveyed key tobacco control informants in the WHO European region between 15 September 2022 and 13 January 2023.

### Participants

Key informants consisted primarily of national WHO FCTC focal points, who are nominated by their country to participate in the official treaty reporting. Contacting them was made possible through assistance from the WHO FCTC Knowledge Hub on Surveillance and Convention Secretariat. In the absence of a functional contact with the focal point (eg, due to personnel changes), other national tobacco control experts were identified with assistance from JATC-2 partners and the WHO European Office for the Prevention and Control of Noncommunicable Diseases. We excluded Switzerland and Monaco due to lack of contacts, and the Russian Federation due to the suspension of research collaboration because of the war in Ukraine. From each country, one coordinated response was requested if the respondent engaged other stakeholders. The questionnaire gathered information and expert opinions on national-level policies and was, therefore, not subjected to an ethics approval. Respondents gave an informed consent on their participation.

Responses were received from 24 of 50 countries (19 of 27 EU Member states), with response rates of 48% across the region and 70% within the EU. The respondents were from Austria, Azerbaijan, Belgium, Cyprus, Czechia, Denmark, Estonia, Germany, Finland, France, Hungary, Ireland, Italy, Lithuania, Luxembourg, the Netherlands, North Macedonia, Norway, Portugal, Serbia, Slovenia, Spain, Sweden and Uzbekistan. The majority of the respondents were officials from health ministries/departments/directorates in the government. One respondent was from the interior ministry, two from national authorities specialised in addictions or substance use and one from a public health institute. Respondents were contacted back in March 2023 for potential updates, which were received from Uzbekistan. The JATC-2 partners could further update the information on new national policies up to May 2024. Partner updates were provided by Belgium, Finland, France, Ireland, the Netherlands, Norway, Slovenia and Spain.

### Questionnaire

The questionnaire assessed the existence of national tobacco endgame goals, their definition, the selected time frame, tobacco or nicotine products covered by the goals and the perceived likelihood of adopting/achieving these goals (from 0=very unlikely to 10=very likely). The reason for the selected response was asked. Furthermore, we inquired about adopted or planned endgame measures and measures to prevent industry interference (table 1). The endgame measures for the questionnaire were identified from earlier reviews.^4 8^ In WP9, harm reduction measures are outside the scope of work and were, therefore, not included in the questionnaire. The measures on tobacco industry interference were derived from screening the recommendations of the WHO FCTC Article 5.3 guidelines. Some additional measures of interest to WP9 partners were also added (marked with * in table 1). The questionnaire and more details of its development are available in the WP9 indicator compendium at www.jaotc.eu.^9^

**Table 1:** Measures included in the JATC-2 WP9 questionnaire

Product-oriented	Regulation of nicotine or pH levels to make tobacco products less or non-addictive Product standards that substantially reduce consumer appeal or remove the most toxic products from the market Ban on flavours Ban on slim cigarettes Ban on combustibles Ban on new types of tobacco products Ban on new tobacco brands, variants or packaging Ban on new non-pharmaceutical nicotine products*
Retail-oriented	Reduction in the number of points of sales Restriction of sales to particular categories of retailers Restrictions of points of sales’ location Increase in the cost of retail licenses or supervisory fees Ban of all promotional relationships between tobacco industry and retailers Financial transition support or other incentives to retailers to give up tobacco sales
Consumer-oriented	Ban on the sale of tobacco products to citizens born in or after a certain year Increase in the legal age of tobacco sales* Smoker’s licence or permit for purchasing tobacco products Prescription to purchase tobacco
Market-oriented	Gradual phase-out-approach on combustibles or other products Quota on tobacco manufacture and imports, which would be regularly reduced under a ‘sinking lid’ Price caps for curtailing industry to set its own retail prices Regulated market model (the marketing of tobacco products in the hands of organisations with goals that are consistent with the overall aims of tobacco control or state takeover of tobacco companies by not-for-profit entity with a public health mandate)
Other innovative measures	Substantial increase in income taxes paid on the profits earned or tobacco supplier profits surtax Performance-based regulation where a public agency sets goals for reductions in smoking prevalence that tobacco companies would be required to meet within a certain period Large fines on tobacco companies based on the quantity of their products consumed by minors with the fines being substantially larger than the revenues gained from sales
Prevention of tobacco industry interference	Interaction between government bodies and the tobacco industry limited only to strictly necessary to enable effective regulation Transparency of the necessary interactions (eg, by public hearings, public notice of interactions or disclosure of the records) Tobacco industry initiatives for tobacco control (eg, industry funded or co-funded public education) not accepted, supported or endorsed Tobacco industry offers for assistance or collaboration in tobacco control legislation or policy drafting not accepted, supported or endorsed A code of conduct for public officials, prescribing the standards with which they should comply in their dealings with the tobacco industry Disclosure of conflict of interests required from all persons involved in setting and implementing public health policies with respect to tobacco control Any person employed by the tobacco industry or any entity working to further its interests not allowed to be a member of any government body, committee or advisory group that sets or implements tobacco control or public health policy Contributions from tobacco industry or front groups to political parties, candidates or campaigns prohibited In case the contributions are not banned, full disclosure on contributions is required No public investments are made in tobacco industry and related ventures

* Additional measures to literature reviews^4 8^ that were of interest to JATC-2 WP9 partners.

### Analysis

We describe adopted goals and measures based on respondent-provided details supplemented with publicly available information on the goals and measures (from, eg, governmental and EU websites). For plans or proposals, we disclose country names only if the information is publicly available to prevent industry interference. We present quotes from the experts’ open-ended responses. This article does not seek to present an exhaustive list of endgame goals and measures in Europe but provides examples and experiences, which can help draw an overview of their status and future directions.

## Tobacco endgame goals

### Official goals adopted or acknowledged by governments

Altogether eight countries reported official tobacco endgame goals (table 2). These were divided into general population goals without subgroup targets and goals including certain generations or subgroups. Most of the countries are aiming for less than 5% prevalence of use, but three countries aim at no use at all in certain subgroups addressing children or pregnant women. Three countries define their prevalence goals specifically as daily use. All countries except Norway have set a target year between 2025 and 2040. The official definitions focus on smoking or tobacco use, except for three countries that also mention nicotine products or tobacco-related products. Some countries extend the scope of endgame compared with the main definition: Belgium and the Netherlands reported including all tobacco and non-pharmaceutical nicotine products, while France and Norway also reported including heated tobacco products (HTPs) under their smoking targets. Finland and Norway have integrated the endgame goal into the objective of the tobacco control law.

**Table 2:** Official tobacco endgame goals among the countries responding to the JATC-2 WP9 questionnaire

Country	Official goal	Specification	Launch year	Target year	Integrated to tobacco control legislation
Belgium	Smoke-free generation^35^	To reduce the number of daily users of tobacco products to 5% in the population aged 15 years and older To reduce the number of people who start using tobacco products to 0% or almost 0%	2022	2040	No
Finland	Ending the use of tobacco and other nicotine products^36^	Less than 5% of the adult population will use tobacco and nicotine products daily (originally 2% of adult population will use tobacco products)	2010 (updated in 2016)	2030 (originally 2040)	Yes: The objective of the law is to end the use of tobacco and other nicotine containing products that are toxic to humans and cause addiction^37^
France	Tobacco-free generation^38^	Children born since 2014 become the first generation of adult non-smokers (<5% smokers)	2018	2032	No
Ireland	Tobacco Free Ireland^39^	Smoking prevalence rate of less than 5% of the Irish population	2013	2025	No
The Netherlands	Smoke-free generation^40^	Fewer than 5% of the residents of the Netherlands aged 18 years and over and 0% of young people and pregnant women will smoke	2018	2040	No
Norway	Tobacco-free society^25^	The proportion of daily smokers and snus users should be below 5% in all age and education groups Children born in 2010 and later must not use tobacco products and related products	2013 (specification in 2023)	–	Yes: The objective of the law is to create a tobacco-free society^41^
Slovenia	Tobacco-free Slovenia^42^	Less than 5% of Slovenia’s population aged 15 or over will use tobacco, related products and other nicotine products that are not registered as nicotine replacement therapy	2022	2040	No
Sweden	Smoke-free Sweden^43^	Less than 5% of the population smokes	2016	2025	No

### Proposals from governmental bodies or other relevant organisations or entities (eg, NGOs, political parties, public health organisations)

Altogether seven countries reported endgame proposals from their countries. In Denmark, the former government introduced a Nicotine-Free Generation goal where no one born since 2010 should start smoking or using nicotine products,^10^ but this proposal has not progressed. A strategy for tobacco-free Germany by the German Cancer Research Center, supported by several NGOs and research entities, aims for <5% adult and <2% adolescent prevalence in tobacco and non-pharmaceutical nicotine use by 2040.^11^ Additionally, the German government’s strategy for the Sustainable Development Goals contains a goal close to the common endgame prevalence level, namely, of 7% smoking prevalence among youth by 2030.^12^ In Italy, scientific societies and independent scientists have allied to advocate for the development of a national tobacco endgame strategy.^13^ In Spain, a new comprehensive plan for the prevention and control of tobacco for years 2024–2027 includes a goal to achieve <5% prevalence of daily use among 14–18-year olds.^14^ Previously, public health organisations and civil society associations published an endgame declaration calling for a goal of <5% smoking prevalence by 2030 and 2% by 2040 in Spain.^15^ Two other countries reported that an endgame proposal exists but is not yet publicly available. One was part of national health strategy discussions, where a goal in line with the EU goal has been proposed. From the second, no details were provided.

## Perceived challenges and opportunities in tobacco endgame

Among the respondents from eight countries with official endgame goals, six provided a score for the likelihood of achieving their goal. On a scale of 0–10, the responses were either 6 (three countries) or 7 (three countries), reflecting moderately positive expectations. Concerns were expressed in relation to non-combustible and new nicotine products, differences between population groups, industry interference, cross-border marketing and sales, sustaining the political will and challenges in estimating the impact of the measures (table 3).

**Table 3:** Respondents’ reflections on the perceived likelihood of achieving their official governmental tobacco endgame goals (six countries, panel A), and on the perceived likelihood of adopting such goals in their country (12 countries, panel B).

Panel A: Achieving the national tobacco endgame goal	Panel B: Adopting a national tobacco endgame goal
‘We are quite confident that for combustible tobacco products we will achieve the goal, but for novel and emerging tobacco and nicotine products we think it is likely that the goal will not be achieved.’ ‘We are on track for smoking but have a problem with smokeless tobacco. The prevalence is high among youth and there is less political will to tackle this problem. It also remains to be seen how the introduction of e-cigarettes on the market will influence the development.’ ‘The challenge is overall nicotine dependency, even though smoking prevalence is decreasing. The challenges are increased use of new nicotine products (like nicotine pouches), possible new tobacco and nicotine products, and use of snus in certain groups.’ ‘Indicators of youth smoking are declining. However, the prevalence among 18–75 year-olds remains high.’ ‘Innovative tobacco industry with large budgets; political will has to be steady in the future; cross-border marketing and sales are hard to beat in practice.’ ‘The implementation of the measures is very likely, however their impact on smoking habits is difficult to predict.’	‘At the moment, there is no government plan or commitment. There is contrary pressure from the tobacco industry aimed at the population but also at the political level.’ ‘A tobacco endgame goal has been demanded by various experts from the prevention and scientific communities, however, currently there is no political majority for it to be adopted.’ ‘In this moment I do not see political support for that idea.’ ‘There is reluctance from the government to implement the law regarding smoking. There will probably be similar reluctance to implement any tobacco endgame proposals.’ ‘The tobacco industry is strong and influential and includes four biggest international tobacco brands operating in the country.’ ‘With commitment from the government and civil society could be achievable, but challenging, because of industry interests.’ ‘Tobacco control is not the priority of the national health policy, which was enforced during the COVID-19 pandemic when all resources were directed to fight pandemics.’ ‘In the near future, there is no intention to prepare a strategy focused on tobacco, as our goal is to reduce the health, social, economic and non-material damages associated with addictive behaviour. Addiction policy focuses on prevention and reduction of harm, not on final goals for specific products.’ ‘We have high prevalence levels so it is a difficult goal.’ ‘Based on the current situation, it is expected that the prevalence of tobacco consumption will increase due to new tobacco and nicotine products and devices.‘ ‘The draft document was approved by all the responsible ministries and the Government. No one expressed doubts about achieving the intended goals.’

The quotes reflect the personal opinions of the respondents. The quotes have been shortened, and translated into English, if necessary.

Among 12 of the 16 countries without an endgame goal who provided a score, the expectations of adopting such a goal in their own country varied greatly: from very negative 0–2 (five countries) and somewhat unsure 5 (three countries) to rather positive 7–8 (two countries). Two countries perceived the adoption very likely, scoring 10. Concerns among these countries related to lack of political will, industry interference and problems in current tobacco control processes, shifting the focus to the COVID-19 pandemic, and the current high use of tobacco and related products (table 3). Some countries reported a preference for general addiction or non-communicable disease (NCD) prevention strategies over tobacco control strategies. Having previously established governmental prevalence reduction goals in a cross-cutting way was seen as a strength for moving towards an endgame approach.

## Tobacco endgame measures

Independently of whether a national tobacco endgame goal exists, a few measures that can contribute to such a goal were already implemented to some extent. These are presented in table 4 according to the taxonomy set in table 1 and summarised below.

**Table 4:** Adopted and planned tobacco endgame measures and forward-looking tobacco control measures among the countries responding to the JATC-2 WP9 questionnaire

Category and measure type	Adopted measures	Planned measures*
Product-oriented		
Product standards	**Menthol fully prohibited in combustibles as an additive that facilitates inhalation**: Belgium, Finland, Germany, Hungary, the Netherlands (from April 2025) **E-cigarette flavour regulation:** Hungary (no flavours allowed), Finland and Norway (only tobacco allowed), Denmark (only tobacco and menthol allowed), Belgium (legislation amended to develop a list of prohibited ingredients), the Netherlands (16 allowed flavour components defined), and Slovenia (similar ban as in the Netherlands) **Advanced plain packaging:** Denmark, Finland and Norway (standardised e-cigarette unit packets), Finland (standardised appearance of individual cigarettes, nicotine e-liquids, and e-cigarette refill containers so that similar category products do not differentiate from each other by shape, colour, surface or other way) **Ban on slim cigarettes:** Hungary, the Netherlands **Partial ban on combustibles:** Norway (prohibition of imports and sales of waterpipe tobacco)	**Menthol prohibited as an additive that facilitates inhalation:** two countries (not specified) **E-cigarette flavour regulation:** Spain^14^, Ireland^44^ **Nicotine pouch flavour regulation:** Finland^45^ **Advanced plain packaging:** France (e-cigarette unit packets),^38^ Denmark (standardising the appearance of individual cigarette),^46^ Norway (health warnings on individual cigarette sticks),^25^ Finland and Denmark (standardised packaging and product appearance for nicotine pouches)^45 46^
Ban on new tobacco or nicotine products	**Authorisation scheme:** Norway (all novel tobacco and nicotine products—all applications for HTPs and nicotine pouches have been rejected thus far), Germany (all novel tobacco products), Portugal (all novel nicotine products) **Ban on disposable e-cigarettes:** Belgium **Ban on nicotine pouches:** Belgium	**Ban on disposable e-cigarettes:** France,^38^ Ireland,^44^ Norway **Ban on nicotine pouches:** the Netherlands (>0,035 mg nicotine/pouch banned since November 2021, now proposed legislation on complete ban)^47^ **Ban on products which do not fall into existing product categories or are placed on the market after a certain date:** one country (not specified)
Retail-oriented		
Reduction in the number of sales points	**Ban on sales in retail types or locations related to minors:** Czechia, France, Ireland, Lithuania, Spain **Stepwise restriction of sales:** the Netherlands (online sales of tobacco and related products prohibited from July 2023, supermarket sales from 2024; legislation being prepared for a sales ban in petrol stations from 2030; only specialist stores will be allowed to sell tobacco and related products from 2032 and e-cigarettes from 2025), Belgium (ban on temporary points of sale including festivals from January 2025, tobacco and e-cigarette sales in large supermarkets (>400 m²) from July 2025) **Restricting sales to specialist shops:** France (tobacco can only be sold by specialist shops), Hungary (tobacco products, e-cigarettes, e-liquids, nicotine pouches, and herbal products for smoking may only be sold by specialist shops and the number of shops is limited to 1 shop/4000 people) **Increased license or supervisory fees:** Finland (high annual supervisory costs to tobacco and e-cigarette retail license holders) **Incentives to give up tobacco sales:** France (a protocol to support tobacco retailers in transforming to other local shops between the confederation of tobacconists and the Ministry of Finance)	**Substantial reduction in retailers:** Norway (in strategy, not yet proposed how)^25^ **Ban on sales in retail types or locations related to minors:** Cyprus (near schools)^48^ **Restricting sales to specialist shops:** One country (e-cigarettes) **Increased license or supervisory fees:** Finland (proposal to increase supervisory fees and ban the granting of a retail license to temporary and mobile sales places)^36^
Consumer-oriented		
Age limits		**Raising the minimum age of sales above 18:** Slovenia (sales to 21 foreseen in the national strategy),^42^ the Netherlands (sales to 21 being investigated by the government),^47^ Finland (sales and possession to 20 proposed by a ministerial working group),^36^ Ireland (sales to 21 proposal for legislation approved),^16^ Norway (proposal only on e-cigarettes to 25 previously in a public consultation),^25^ one country (not specified) **Tobacco- and nicotine-free generation:** Norway (those born in 2010, decision and details on implementation open)^25^
Other		**Prohibiting the import, purchase and possession of nicotine products that are illegal to market in the country:** Denmark^46^
Market-oriented		
Price caps	**Curtailing tobacco industry to set its own retail prices:** Sweden (each year, the producers or the importer must set a retail price for the product, which is used to calculate an excise duty even if the product is sold cheaper. If retailers sell the product at a higher price, they pay additional excise duty based on the actual selling price)	
Regulated market model	**State monopoly on tobacco sales:** Austria, France, Hungary, Italy and Spain	

* Status of planning includes such measures that are under political consideration either already as public (eg, in consultation) or in preparation but not yet public. Available details are given in the text.

### Product-oriented measures

The EU Tobacco Products Directive (TPD) and the delegated directive 2022/2100 prohibit characterising flavours in cigarettes, roll-your-own and HTPs, but some countries go beyond this to reduce product appeal with different product standards. Five countries had fully prohibited menthol as an additive that facilitates inhalation in combustibles, and seven countries had prohibited all or most flavours in e-cigarette liquids (table 4). These measures were also planned in some countries, and Finland was processing regulation on nicotine pouch flavours. Plain packaging had been extended from tobacco products to e-cigarette packaging in three countries and was also considered for nicotine pouch packaging in two. Some countries had standardised or were standardising the appearance of individual cigarettes, nicotine e-liquids, e-cigarette refill containers and/or nicotine pouches. Health warnings on individual cigarette sticks were considered in Norway, which had also prohibited imports and sales of waterpipe tobacco, therefore partially addressing a ban on combustibles.

The TPD allows Member States to prohibit a certain category of tobacco or related products if the Commission approves it after considering whether national provisions are justified, necessary and proportionate, and whether they constitute a disguised barrier to trade. Belgium has received approval to prohibit disposable e-cigarettes, and two other countries also have proposals to introduce such a ban. Two countries reported an authorisation scheme for novel tobacco products, where the government authorises or rejects market entry applications. Non-pharmaceutical nicotine products (other than e-cigarettes) are not under TPD and countries regulate their market entry independently. An authorisation scheme for novel nicotine products was reported by two countries. Two countries have prohibited nicotine pouches. One country reported considering a ban on products that do not fall into existing product categories or are placed on the market after a certain date, but no specification was available.

### Retail-oriented measures

Some countries reported prohibiting or restricting tobacco or related product sales in retail types or locations related to minors, and Cyprus was planning to restrict points of sales near schools (table 4). Broader restrictions were still rare. New stepwise sales reductions were adopted in two countries, and a substantial reduction in retailers was set to the strategy in Norway but without concrete proposals. Two countries limited tobacco sales to specialist shops, and one country was considering including also e-cigarette sales to these. Hungary has set numerical limits to the density of the specialist tobacco shops. Finland introduced high annual supervisory fees to retail license holders and has had a proposal to prohibit the granting of a retail license to temporary and mobile sales places. France supported the transition of tobacco retailers into other local shops and no longer selling tobacco.

### Consumer-oriented, market-oriented and other innovative measures

Most of the proposed endgame measures in these categories were not in place or planned. Plans focused on consumer-oriented measures, mainly age limits of 20 or 21 years, where altogether six countries have had proposals to raise the age of sale above 18. Of these, Ireland already approved in May 2024 a proposal for legislation that will increase the age of sale of tobacco to 21, aiming to be the first EU country to do so.^16^ In Norway, a tobacco-free and nicotine-free generation to those born in 2010 is envisaged in a national strategy, but decisions and details on its implementation are awaited. In Denmark, the new prevention agreement proposes prohibiting the import, purchase and possession of nicotine products that are illegal to market in the country. Sweden is utilising excise duty for curtailing industry to set its own retail prices. Five countries have a regulated market model where the state has a monopoly on tobacco sales.

## Preparedness to counter tobacco industry interference

While many respondents referred to implementing Article 5.3 of the WHO FCTC, concrete tools to prevent industry interference were often missing. However, some examples of adopted measures were shared. These addressed legislative measures, lobbying registers, a code of conduct/procedure, public disclosure of necessary correspondences, disclosure of lobbying expenses, plans to better regulate production and industry reporting obligations, and ethical guidelines preventing state investments in the tobacco industry (table 5). As for planned measures, three countries were developing guidelines on contact between the industry and governmental organisations, one country was planning to develop a transparency register of contacts between the tobacco industry and government, and another country for the disclosure of the records from necessary interactions.

**Table 5:** Regulations and measures to prevent tobacco industry interference among the countries responding to the JATC-2 WP9 questionnaire

Country	Measure(s)
Finland	Integrating the principles of Article 5.3 into the Tobacco Act objective has been proposed by a ministerial working group. A Finnish Transparency Register for lobbyist has been opened.
France	Manufacturers, importers and distributors of tobacco products as well as entities representing them are required to declare to the Ministry of Health all expenses linked to activities for the representation of their interests and this information is published at the ministry's website.
Ireland	All lobbyists are required to register and make a return of activities every 4 months.
Lithuania	Law obligates to protect against interests of the tobacco industry when implementing policies related to tobacco control.
The Netherlands	A code of conduct has been published online. All the necessary correspondence with the tobacco industry is publicly disclosed, and the government produces regularly a letter to remind ministries of Article 5.3.
Norway	National strategy includes plans to better regulate the production of tobacco products, following the entrance of snus manufacturing in the jurisdiction, and to expand the reporting obligations of the tobacco industry and better implement the Article 5.3. The ethical guidelines of the Norwegian State Pension Fund prohibit investments in the tobacco industry.
Serbia	The Code of Procedure of the National Committee for Tobacco Control requires a conflict-of-interest statement from each member.
Slovenia	Several Acts oblige civil servants not to engage in activities with conflict of interest or gainful activities. Any form of contribution to an event, activity or individual with the aim or possible effect of promoting tobacco and related products or their use is prohibited.

## Discussion

Our results indicate both momentum and challenges in adopting and disseminating measures that facilitate achieving the EU Tobacco-Free Generation goal of less than 5% tobacco use by 2040. Almost half of the 27 EU Member States either have already adopted a national tobacco endgame goal or have a proposal for such a goal from the government, civil society or research entities. Outside the EU in the WHO European Region, Norway reported an official tobacco endgame goal. While most of the countries with an official goal aim for a similar <5% prevalence level as the EU goal, the definitions of goals and their specifications in the government documents vary considerably. For some countries, this can also pose challenges in measuring the progress. In Ireland and Sweden, the target year of 2025 is approaching soon, calling for the first comprehensive evaluations of national tobacco endgame strategies in the region. Including tobacco endgame as an objective of tobacco control legislation—like in Finland and Norway—may provide sustainability behind changing governmental programmes or strategies and political will.

In the EU, the Member States have benefitted from common minimum product standards set in the TPD. While several countries already go beyond the TPD to address attractiveness and appeal, no measures that would substantially reduce addictiveness were adopted or planned. To meet the <5% prevalence level by 2040, the TPD should be developed from this perspective in a forward-looking way. The EU has invested substantial effort and resources into the advisory mechanism for the prohibition of characterising flavours.^17^ Yet a simplified, effective approach would be to follow the WHO FCTC Article 9 and 10 guidelines to prohibit the use of all ingredients that make tobacco products attractive, including flavouring agents. Furthermore, the EU-level nicotine limits for cigarettes could be lowered to make them less or non-addictive, leading to their gradual phase-out from the market. Based on the evidence, reducing nicotine content in cigarettes to very low levels could improve public health and have benefits across different population groups by decreasing the uptake of regular smoking, decreasing the amount smoked and increasing smoking cessation.^18^ Introducing very low nicotine cigarettes on the EU level could be a balanced and justified measure considering the increased product supply caused by the continuous entrance of novel tobacco or nicotine products to the market. These novel products were mainly seen as challenges in tobacco endgame by the respondents, and several countries are already covering nicotine products such as e-cigarettes and nicotine pouches in their endgame goals or measures. This can be seen as a forward-looking approach to respond to tobacco industry strategies, which aim to increase product portfolio and profit, attract new customers and delay and distract from effective control policies.^19^ Clear separation between measures to only reduce harm and measures to end the tobacco epidemic may help regulators and policymakers to understand and identify measures that are feasible and likely to produce substantial impact in their local context.

The reported retail-oriented and consumer-oriented measures tended to focus on reducing the sales points by limiting sales to certain retailers and raising the age limit of sales above 18 years. For example, substantial stepwise reductions in retail outlets are beginning to be implemented in the Netherlands and in Belgium. Yet, most countries in the region would still need to introduce retail licensing to effectively control and reduce retail density.^20^ In Finland, the licensing with high annual costs has gradually reduced the number of tobacco retailers to approximately a half. However, the number remains high and unequally distributed to more socioeconomically disadvantaged areas—reminding of the continued need to consider the impact of tobacco endgame measures in different population groups.^21^ In Hungary, the introduction of state-owned specialist tobacco shops has decreased the density of tobacco shops by 85%, concurring with declining adolescent smoking.^22^ The age limits that were under consideration focused on 20 or 21 years. In the European context, where no country yet has implemented an age limit above 18 years for tobacco, this measure could have a substantial impact considering most of the initiation occurs by the age of 20.^23 24^ In Europe, Norway was first to publish in March 2023 a goal that children born since 2010 do not use tobacco and nicotine products, but its practical implementation is undecided.^25^ The United Kingdom has then moved ahead by announcing in October 2023 that it will become an offence to sell tobacco products to anyone born on or after 1 January 2009.^26^ Based on the evidence, the retail- and consumer-oriented measures, especially if combined, can be expected to have a notable impact on tobacco use prevalence and lead to health gains over time.^23 27^

The EU goal can be used to support the development of similar national goals. Additionally, it can be used to bring the need for better implementation of the WHO FCTC to the political agenda, connected to the national work for NCD prevention and sustainable development goals. This can be beneficial especially in countries where adopting an endgame goal is not yet seen as feasible in the current tobacco control context. The implementation of the WHO FCTC as well as the capacity for tobacco control needs to be strengthened in Europe.^28^ As part of this, countries should look into measures that tap into tobacco industry profits, which are mostly not even planned in the region. Together with the lack of concrete tools to prevent and counter industry interference, this enables the industry to mobilise resources for lobbying and distracting policymaking away from timely and effective measures. Industry interference was identified as a challenge both in adopting and achieving tobacco endgame goals. Better protection is needed even on the EU level, as shown in the recent European Ombudsman investigations.^29^ Besides national actions, the EU-level investment and support for the enforcement of tobacco control, together with the regular revision of key directives and recommendations, are essential for achieving the EU goal. An interesting comparison can be found in food safety where the EU audits the application and effectiveness of the laws and controls and provides training to the responsible authorities.^30^

Finally, the EU goal can be used to raise awareness of the tobacco endgame approach, leveraging support from civil society and the public. For instance, a study from Ireland showed low awareness but broad support for the local tobacco endgame goal.^31^ In the Netherlands, key factors in accelerating tobacco control have been the genesis of a ‘Smoke-free Generation’ movement in the wider society, initiated by the three main national charities, combined with stricter adherence to Article 5.3 of the WHO FCTC and a comprehensive marketing ban.^32^ In 2022, several European civil society associations launched a joint European Citizen’s Initiative calling for a broad range of measures including tobacco-free environments and ending the sale of tobacco and nicotine products to citizens born since 2010, but it did not reach enough signatories.^33^ To facilitate the dissemination of measures that are likely to have a substantial impact within a reasonable timeframe, knowledge sharing between countries with different tobacco control contexts and approaches is needed. Multinational collaborations such as the JATC-2 can serve as platforms to share best practices and act as vehicles to overcome the barriers of lack of knowledge or political will. A great global opportunity for information exchange presents in the 11th session of the Conference of the Parties of the WHO FCTC in 2025, where an expert group established by the COP10 will present its report on Article 2.1 and forward-looking tobacco control measures.^34^ The possibility of shifting the focus from controlling to ending the tobacco epidemic is an important message to convey to policymakers.
